# Novel glycolipid agents for killing cisplatin-resistant human epithelial ovarian cancer cells

**DOI:** 10.1186/s13046-017-0538-9

**Published:** 2017-05-12

**Authors:** Amani I. Moraya, Jennifer L. Ali, Pranati Samadder, Lisa Liang, Ludivine Coudière Morrison, Tamra E. Werbowetski-Ogilvie, Makanjuola Ogunsina, Frank Schweizer, Gilbert Arthur, Mark W. Nachtigal

**Affiliations:** 1 Dept. of Biochemistry & Medical Genetics, University of Manitoba, Room 333 BMSB, 745 Bannatyne Avenue, Winnipeg, R3E 0 W9 MB Canada; 2 Dept. of Chemistry, University of Manitoba, Winnipeg, Canada; 3 Dept. of Obstetrics, Gynecology & Reproductive Sciences, University of Manitoba, Winnipeg, Canada; 4 Research Institute in Oncology & Hematology, CancerCare Manitoba, Winnipeg, Canada; 5 Manitoba Ovarian Cancer Outcome (MOCO) study group, Winnipeg, Canada

**Keywords:** Ovarian cancer, Drug-resistance, Glycosylated anti-tumour ether lipid, Spheroid

## Abstract

**Background:**

Chemotherapy resistance is one of the major factors contributing to mortality from human epithelial ovarian cancer (EOC). Identifying drugs that can effectively kill chemotherapy-resistant EOC cells would be a major advance in reducing mortality. Glycosylated antitumour ether lipids (GAELs) are synthetic glycolipids that are cytotoxic to a wide range of cancer cells. They appear to induce cancer cell death in an apoptosis-independent manner.

**Methods:**

Herein, the effectiveness of two GAELs, GLN and MO-101, in killing chemotherapy-sensitive and –resistant EOC cells lines and primary cell samples was tested using monolayer, non-adherent aggregate, and non-adherent spheroid cultures.

**Results:**

Our results show that EOC cells exhibit a differential sensitivity to the GAELs. Strikingly, both GAELs are capable of inducing EOC cell death in chemotherapy-sensitive and –resistant cells grown as monolayer or non-adherent cultures. Mechanistic studies provide evidence that apoptotic-cell death (caspase activation) contributes to, but is not completely responsible for, GAEL-induced cell killing in the A2780-cp EOC cell line, but not primary EOC cell samples.

**Conclusions:**

Studies using primary EOC cell samples supports previously published work showing a GAEL-induced caspase-independent mechanism of death. GAELs hold promise for development as novel compounds to combat EOC mortality due to chemotherapy resistance.

**Electronic supplementary material:**

The online version of this article (doi:10.1186/s13046-017-0538-9) contains supplementary material, which is available to authorized users.

## Background

Chemotherapy resistance of epithelial ovarian cancer (EOC) cells is a major contributor to reducing the survival rate among EOC patients [1–3]. EOCs are typically treated by surgically debulking the pelvic disease and with chemotherapy [2]. While the majority of patients respond to initial chemotherapy usually comprising 6–9 cycles of a platinum agent (carboplatin) and a taxane, up to 75% of EOC patients will relapse within 18 months with chemotherapy-resistant disease [4, 5]. Therefore, there is an unmet clinical need to improve the treatment of patients with recurrent, drug resistant EOC.

Chemoresistance is defined clinically by disease recurrence less than six months after initial treatment. Recurrent disease is treated with drugs such as gemcitabine, liposomal doxorubicin, or topotecan, which have been shown to increase progression-free survival by 10-30% in platinum-resistant EOC [2, 6]. Presently, recurrent EOC is essentially an incurable disease. Thus, evaluating novel drug treatments using models of chemotherapy resistant disease becomes critical to improve outcome for this patient population.

Monolayer culture systems have provided a wealth of information about cancer cell biology, but there is recognition of the limitations of this system and its relevance to in vivo cell biology [7–9]. Although, the majority of the literature has used the two-dimensional monolayer system as a model for testing anti-cancer therapies, the success of treatment in clinical trials is only approximately 5% [10]. While the monolayer system often shows promising results with experimental compounds in vitro, these activities often fail in vivo. Three-dimensional or non-adherent culture systems are considered more representative of in vivo conditions, typically using culture plastics that do not promote cell adhesion (non-adherent cultures) or hanging droplet techniques resulting in loose cell aggregates or spheroid formation [7–9, 11–13]*.* Importantly, cells in non-adherent cultures exhibited frequently higher levels of chemoresistance compared to adherent conditions [9, 14–16]. Testing drug treatments using these systems will likely decrease the gap between laboratory research and clinical trials.

Current efforts to kill EOC cancer cells is mostly based on damaging the DNA, preventing DNA synthesis, or targeting the cell cycle to stop cell proliferation. Targeting these biological events activates apoptotic pathways that induce cell death. However, EOC cells are either inherently resistant or capable of developing resistance during chemotherapy treatment by various pathways to evade apoptosis [3, 12, 17–22]. Glycosylated antitumour ether lipids (GAELs) are synthetic small molecular weight amphiphilic glycolipids that are cytotoxic to a wide range of cancer cells [23, 24]. However, there has been little research investigating the effects of GAELs on EOC cells [25]. The prototypical GAEL, containing a 2-amino-glucose head group (GLN; 1-O-hexadecyl-2-O-methyl-3-O-(2’-amino-2^’-^deoxy-β-D-glucopyranosyl)-sn-glycerol) kills cells by an apoptosis-independent mechanism [26, 27]. GAELs exhibited a distinct mechanism of action from other antitumour ether lipids and current anti-cancer agents, and there is evidence GAELs enter cancer cells via an endocytic pathway, which leads to the generation of large acidic vacuoles and the release of acid hydrolases, including cathepsin D, that induce a caspase-independent form of cell death [23, 28]. We have recently reported that GAELs not only inhibited spheroid formation by tumour propagating cells derived from breast cancer cell lines, but they also caused the disintegration of tumour propagating cell spheroids and killed the cells [28].

There are few reliable models of drug-resistant EOC cells available for research [7, 12, 29]. The two most popular are (A) A2780-s (sensitive) and A2780-cp (cisplatin resistant) isogenic cell lines representing the endometrioid subtype of EOC [30], and (B) PE01 and PE04 cells established from the ascites of a patient with poorly differentiated serous adenocarcinoma before/after development of clinical resistance. As an alternative to these immortalized cell lines, investigators use primary EOC cell samples isolated from solid tumours or ascites before and after manifestation of clinical resistance. Herein, we used the A2780-s/A2780-cp cell lines and primary cells from seven different EOC patients to test the effect of GAELs on EOC cell viability. Moreover, the GAEL effects were tested on cells grown as adherent monolayers, and non-adherent cellular aggregates or spheroids. Experiments were performed to study the effect of GLN and the most active GAEL we have synthesized to date, 1-O-Hexadecyl-2-O-methyl-3-O-(2’,6’-diamino-2’,6’-dideoxy-a-D-glucopyranosyl)-sn-glycerol (MO-101), on the cell viability of these different platinum-resistant models of EOC. The differences between the two structurally similar GAELs is the presence of two cationic (NH2) groups in MO-101 while GLN has only one cationic group. As GLN and MO-101 effectively killed platinum-sensitive and platinum-resistance EOC cells, investigations were conducted to provide insight into putative mechanisms of action for these drugs in EOC cells. Our results support further investigation of GAELs as novel agents for the treatment of recurrent, platinum-resistant ovarian cancer.

## Methods

*Cell Culture.* Primary human EOC cells were isolated from ascites fluid obtained from patients with ovarian adenocarcinoma (for patient characteristics, see Additional file 1: Table S1), and grown as previously described [31, 32]. All experiments with primary EOC cells were performed between passages 1 and 3. The A2780-s and A2780-cp endometrioid EOC cell lines were obtained from Dr. B. Tsang (University of Ottawa), and were authenticated by short tandem repeat profiling in June 2016 using the Promega PowerPlex system (ATCC cell authentication service). The COV362 cell line (passage 36; ECACC catalog # 07071904) was purchased from Sigma-Aldrich. Cells were maintained without antibiotics in DMEM/F12 + fetal bovine serum (10% v/v). For experiments with non-adherent cultures, cells were seeded from adherent cultures into ultralow attachment plates (Greiner Bio-One CELLSTAR® Cell-Repellent Surface Microplate # 655970) for 48 h (h) prior to drug treatment. All cells were maintained at 37 °C, 5% CO_2_/95% air, 100% humidity.

*Drugs.* Glycosylated antitumor ether lipids (GAELs), specifically GLN and MO-101 (compound 2) were synthesized as described in Ogunsina *et. al* [33]. Structurally GLN and MO-101 differ by introduction of an amino substitution at the C_6_-position of glucose and the nature of the anomeric linkage (Additional file 2: Figure S1). Cisplatin (Tocris Bioscience), Q-VD-OPh (pan-caspase inhibitor, APExBio), and staurosporine were reconstituted in 100% dimethyl sulfoxide (DMSO). The GAELs and pepstatin A (acid protease/cathepsin D inhibitor) were reconstituted in 95% ethanol. DMSO and ethanol were used as vehicle controls where applicable.

*Cell viability.* *Adherent cultures*: Cells were seeded at 4000 cells/well in 96-well plates and grown for 24 h before adding drugs. Cells were treated with cisplatin (DMSO vehicle control) or with GAELs (95% ethanol vehicle control) for the times indicated. The concentrations of ethanol did not exceed 0.1% (v/v). *Non-adherent cultures*: Cells were seeded at 10 000 cells/well in 96-well ultralow attachment plates and allowed to form aggregates or spheroids for 48 h prior to drug treatment.

To assess viability, Cell Titer 96 Aqueous One Solution Reagent (Promega Corporation, G3580), was added to each well and incubated for 1–4 h at 37 °C. Absorbance was read at 490 nm with a SpectraMax M2^e^ (Molecular Devices) and the quantity of formazan product being directly proportional to the number of viable cells.

*Caspase-Dependent Cell Death Assay.* Cells were plated in either 96-well or 24-well ultralow attachment plates and allowed to grow for 48 h prior to drug treatment. Cells were pre-treated for 4 h with the pan-caspase inhibitor Q-VD-OPh (QVD; 25 μM) followed by addition of MO-101, GLN or cisplatin with the inhibitor for 48 h. Cells were analyzed for viability using Cell Titer 96 Aqueous One Solution Reagent or by flow cytometry using the CaspGLOW Fluorescein Active Caspase Staining kit (BioVision Inc., K180-100) to detect activated caspases. For these experiments, additional cell viability data was determined by trypan blue exclusion and measured using a BioRad TC-20 Automated Cell Counter.

*Flow cytometry measurement.* Cell viability was assessed using an APC Annexin V staining kit (BD Pharmigen, 559763). A2780cp cells were plated into 24-well ultralow attachment plates and allowed to grow for 48 h prior to drug addition. Cells were treated with MO-101 (7.5, 12.5 or 15 μM), GLN (15, 20, or 25 μM), or vehicle control for 48 h. Cells were dissociated with Accutase and a single cell suspension at a density of 1x10^5^ cells/ml was stained simultaneously with Annexin V-APC and 7-amino actinomycin D (7-AAD) using concentrations of 1 μl/100 000 cells and 2 μl/100 000 cells, respectively. Samples were incubated for 15 min and then resuspended in 400 μl of 1× Binding Buffer. Flow cytometry was performed on a Gallios™ flow cytometer and analyzed using Kaluza analysis software (Beckman Coulter, Inc.).

To assess cell cycle, A2780cp cells were plated into 24-well ultralow attachment plates and allowed to grow for 48 h prior to drug addition. Cells were treated with MO-101 (5, 7.5, 12.5 or 15 μM), GLN (10, 25 μM), or vehicle control for 48 h. Prior to harvesting, cells were pulsed for 3 h at 37 °C with 1 mM bromo-deoxy-uridine (BrdU, 10 μM). BrdU was detected with anti-BrdU-APC using the APC BrdU Flow Kit (BD Pharmingen, 552598) according to manufacturer’s guidelines. Cells were then counterstained with 7-AAD and flow data acquisition was performed on the MoFloXDP (Beckman Coulter, Inc.). 400 000 cells per condition were analyzed from a minimum of three independent experiments.

*Statistical Analyses.* One way ANOVA with Dunnett’s multiple comparison test were conducted for drug treatment experiments. Unless otherwise stated, the *p* values represent data from three independent experiments conducted in sextuplicate. Flow cytometry data represents three independent experiments.

## Results

### Cisplatin sensitivity of adherent vs. non-adherent EOC cultures

While cell spheroids may be observed in malignant ascites, our laboratory more frequently observes single EOC cells and loose cell aggregates (Additional file 3: Figure S2). The model used for the non-adherent culture experiments conducted herein comprise a model system of cellular aggregates, although some cell samples efficiently form spheroids under these conditions, e.g. EOC013F and EOC061 (Additional file 4: Figure S3). The A2780-s (cisplatin sensitive) and A2780-cp (cisplatin resistant) cell lines, in addition to several primary EOC cell samples (Additional file 1: Table S1), were grown as adherent monolayers, non-adherent aggregates or spheroids. Cell viability in response to cisplatin or two different GAEL compounds, GLN or MO-101, was assessed.

**Fig. 1:**
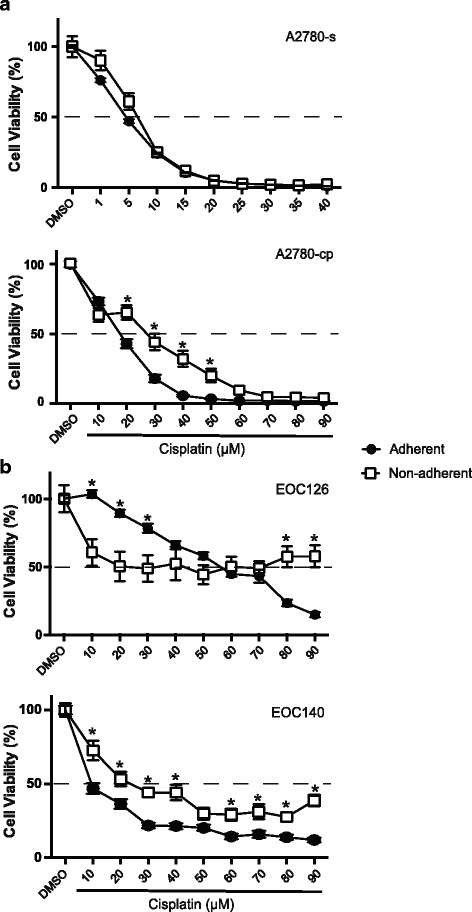
Cisplatin sensitivity of EOC cells grown as adherent (*closed circle*) or non-adherent (*open square*) cultures. **a** Cisplatin dose–response *curves* for cisplatin-sensitive A2780-s and isogenic cisplatin-resistant A2780-cp cells. **b** Cisplatin dose–response *curves* for two different primary EOC patient samples, EOC126 and EOC 140. For A and B cells were exposed to drug for 72 h. Intersections with the *dashed line* approximates the CC_50_ value. Cisplatin doses where there is a significant difference in cell viability between cells grown as adherent or non-adherent cultures are indicated by an asterisk (*), *p* < 0.05

A2780-s cells were quite sensitive to cisplatin treatment (CC_50_ ~ 5-7 μM) with little difference in the cisplatin sensitivity between the cells grown in adherent or non-adherent conditions (Fig. 1a). Concentrations of 25 μM or more resulted in complete inhibition of cell viability. As expected, A2780cp cells were more resistant to cisplatin than A2780s. In contrast to the results observed with A2780s, A2780-cp cells grown as non-adherent aggregates were more resistant to cisplatin than cells grown as monolayer cultures when treated with 20–50 μM cisplatin. The differences were statistically significant (*p* < 0.05). The rightward shift in the CC_50_ concentration changed from ~18 μM to 27 μM.

The effect of cisplatin on viability was also investigated with the COV362 HGSOC cell line grown under adherent and non-adherent conditions. The results revealed a profile that was somewhat similar to the cisplatin sensitivity profile obtained with the A2780cp cells. Greater resistance to cisplatin was observed under non-adherent conditions, and a more dramatic shift in the CC_50_ from ~11 μM to ~36 μM (Additional file 5: Figure S4). At the highest concentration of cisplatin tested, 90 μM, 25% of the cells were still viable. Thus, these cells are more resistant to cisplatin than A2780cp cells.

The effects of cisplatin on the viability of primary cells isolated from EOC patient samples was also investigated (Fig. 1b). In EOC126 (clear cell histotype) grown as adherent cultures, a dose-dependent effect of cisplatin on the viability of the cells was observed with an CC_50_ ~ 57 μM and viability down to 15% at 90 μM. By contrast, in EOC126 cells grown as non-adherent cultures, there was an initial response to cisplatin with cell viability down to 50% at a concentration of 20 μM. Further increases in cisplatin up to 90 μM did not result in any further decrease in cell viability, but compared to monolayer cultures, non-adherent cultures exhibited a statistically significant difference in viability at 80–90 μM. Similarly, cell death in EOC140 cells (HGSOC histotype), which were obtained from a patient with recurrent, platinum-resistant disease, was dose-dependent up to 30 μM using either culture condition, but further increases in cisplatin did not affect cell viability up to 90 μM (Fig. 1b). Statistically significant differences in EOC140 cell viability between the adherent and non-adherent cells were observed with the cells grown under non-adherent conditions being more resistant to cisplatin. Additional examples of differential cisplatin sensitivity when primary HGSOC cells (EOC 146, EOC183A, EOC183I) were grown as adherent or non-adherent aggregates are shown in Additional file 5: Figure S4.

### EOC cell response to GAEL treatment

**Fig. 2:**
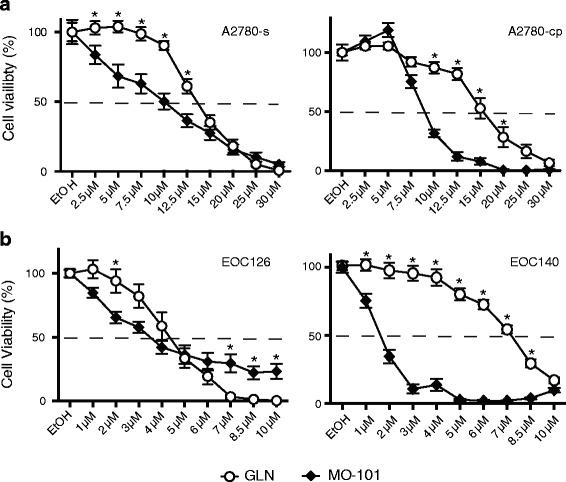
GAEL sensitivity of EOC cells grown in the presence of GLN (*open circle*) or MO-101 (*closed diamond*). **a** Dose–response *curves* for A2780-s (cisplatin sensitive) and A2780-cp (cisplatin resistant) cells. **b** Dose–response curves to GLN or MO-101 for two different primary EOC patient samples, EOC126 and EOC140. For A and B cells were exposed to drug for 48 h. Intersection with the *dashed line* approximates the CC_50_ value. Doses where there is a significant difference in cell viability between cells grown in the presence of GLN or MO-101 are indicated by an asterisk (*), *p* < 0.05

The EOC cell lines grown as non-adherent cultures showed dose-dependent decreases in cell viability when treated with either GLN or MO-101, with MO-101 typically having greater efficacy (Fig. 2). Cisplatin-sensitive A2780-s cells showed reduced cell viability in response to both GAELs (GLN CC_50_ ~ 14 μM; MO-101 CC_50_ ~ 10 μM), but GLN was less effective than MO-101 at killing the cells at concentrations between 2.5-12.5 μM. At doses above 12.5 μM, both GAELs had a similar effect on cell viability. The viability of cisplatin-resistant A2780-cp cells was also decreased with increasing concentrations of GLN and MO-101, with CC_50_ ~ 15 μM for GLN and a CC_50_ ~ 9 μM for MO-101. Similar to the observations with A2780s cells, MO-101 was more effective in killing the A2780-cp cells than GLN. Parallel studies using the COV362 HGSOC cell line showed resistance to GLN at doses up to 15 μM, but sensitivity thereafter (Additional file 6: Figure S5). By contrast, COV362 cells were sensitive to MO-101 at doses higher than 2.5 μM.

The effects of the GAELs on the viability of primary EOC cells isolated from patients was also investigated (Fig. 2 and Additional file 6: Figure S5). We observed that much lower doses of the GAELs were required to kill the cells from primary patient samples (EOC126 and EOC140; Fig. 2) compared to the A2780 cell lines. EOC126 cells that exhibited cisplatin resistance (Fig. 1) were effectively killed by GLN with a CC_50_ value of 4.3 μM. MO-101 was surprisingly unable to kill all the EOC126 cells at the highest concentration tested (10 μM). A CC_50_ value of 3.5 μM was obtained for MO-101, but at 10 μM ~20% of the cells were still viable. By contrast, EOC140 cells were effectively killed by MO-101, but showed some resistance to GLN-induced death. Additional examples of differential primary EOC cell (EOC 146, EOC183A, EOC183I) GAEL sensitivity are shown in (Additional file 6: Figure S5).

**Fig. 3:**
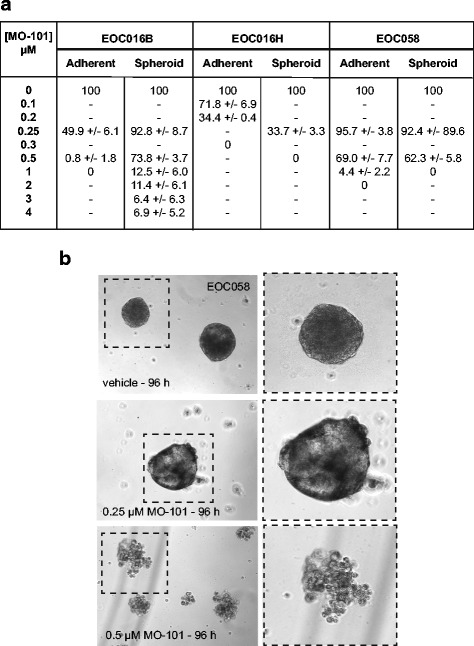
GAEL sensitivity of EOC cells grown as spheroids. **a** MO-101 dose–response of primary EOC cell samples (EOC016B and H, EOC058). Cells were exposed to MO-101 for 72 h prior to determining cell viability. Data is shown as percent viability compared to vehicle treated controls. **b** Effect of MO-101 on EOC058 spheroid integrity after 96 h exposure. The *right-hand panels* show higher magnification images of the spheroids outlined by *dashed boxes* in the *left panel*

To extend these studies, primary cells were grown as adherent monolayer or spheroid cultures, and their response to MO-101 was compared (Fig. 3). Serial samples from the same patient were obtained prior to (EOC016B - chemonaive) and after (EOC016H – platinum resistant) chemotherapy treatment. MO-101 effectively killed these samples and sample EOC058 (HGSOC; chemonaive) in a dose-dependent fashion, even when grown as spheroids (Fig. 3a). As well as assessing the viability of the cells, we also looked at the effect of the GAELs on the integrity of the spheroids. GAELs were able to cause the disintegration of the spheroids and a representative image of the disintegration of the spheroid from EOC058 cells in response to MO-101 treatment is shown (Fig. 3b). Similar results were obtained with two additional EOC samples (EOC013F, EOC061; Additional file 4: Figure S3).

These experiments showed that different patient samples exhibited differential sensitivity and cell proliferation responses to the GAEL compounds. Importantly, these results showed that GAEL compounds can effectively kill cisplatin-sensitive and cisplatin-resistant EOC cell lines and primary cells isolated from patient samples grown as adherent monolayers, aggregates, or spheroids using non-adherent culture conditions.

### Evaluating potential mechanisms of GAEL-induced EOC cell death

**Fig. 4:**
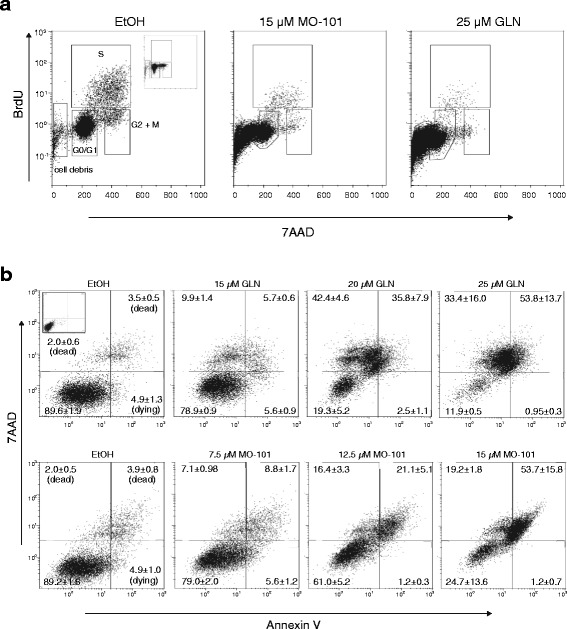
Effect of GAEL to induce cell death. **a** BrdU/7AAD analysis of A2780-cp non-adherent cultures treated with GLN or MO-101 relative to ethanol (EtOH) controls after 48 h. Data corresponding to cells at different stages of the cell cycle are indicated, as are dead (apoptotic; cell debris) cells. **b** Annexin V/7AAD analysis of A2780-cp non-adherent cultures treated with increasing doses of GLN or MO-101 relative to EtOH controls after 48 h. Percentage of cells in each quadrant are indicated

To gain insight into how GAELs may be affecting EOC cell viability, cell cycle analysis by flow cytometry was conducted for A2780-cp cells using BrdU and 7AAD. We observed a shift towards sub-G0/G1, as cells exited the cell cycle in response to GAEL treatment and displayed an increase in cell death (Fig. 4a). In support of this, Annexin V/7AAD staining was conducted to measure A2780-cp cell death after GAEL treatment (Fig. 4b). A2780-cp cells exhibited a dose-dependent increase in dying and dead cells in response to GLN and MO-101 treatment for 48 h, with the highest doses of GLN (25 μM) and MO-101 (15 μM) resulting in ~86% (upper quadrants, 33.4% + 53.8%) and 70% (19.2% + 53.7%) cell death, respectively.

### Pepstatin A did not rescue GAEL-induced EOC cell death

**Fig. 5:**
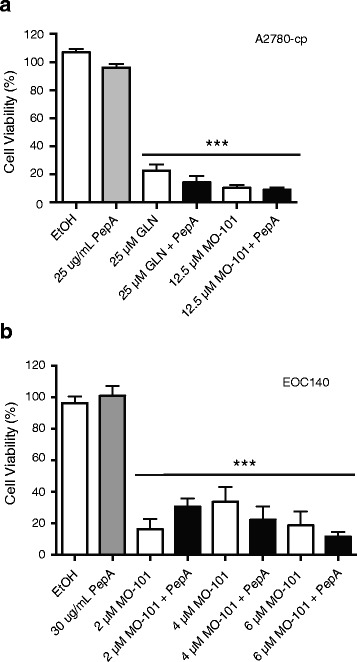
Evaluating the ability of pepstatin A (PepA) to block GAEL-induced cell death. **a** A2780-cp cells were treated with GLN or MO-101 alone or co-treated with 25 μg/mL PepA for 48 h. While GAEL or GAEL + PepA treatment induced a significant level of cell death, there was no statistical difference in the presence of PepA. **b** EOC140 cells treated with increasing doses of MO-101 alone or co-treated with 30 μg/mL PepA for 48 h. There is a statistically significant inhibition of cell death in the presence of PepA for two of the conditions, (+), *p* < 0.05. For both A and B, doses where there is a significant difference in cell viability between cells grown in the presence of drugs compared to vehicle control or PepA alone are indicated by asterisks (***), *p* < 0.001

Previous research demonstrated that GAELs induced an apoptosis-independent form of cancer cell death that may involve altering lysosomal permeability to allow the release of acid proteases such as cathepsin D [26]. GLN-induced cell death could be reduced, but not abrogated, by incubation of cells with the acid protease inhibitor pepstatin A. To investigate if a similar mechanism may operate in EOC cells, A2780-cp cells were incubated with GLN, MO-101, or the GAELs with pepstatin A. The results obtained revealed that neither GLN- or MO-101-induced cell death were attenuated by pepstatin A co-treatment (Fig. 5a). These data suggest that acid proteases such as cathepsin D do not play a role in GAEL-induced cell death in A2780-cp cells. The involvement of other cathepsins were not investigated and cannot be ruled out. A similar response was observed in EOC140 and EOC183A cells treated with MO-101 (Fig. 5b and Additional file 7: Figure S6a). By contrast, pepstatin A was capable of reducing the cell killing effects of MO-101 in the primary EOC146 cell sample at some dose combinations (Additional file 7: Fig. 6b). In particular, protection was noted with the combination of 5 μM MO-101 + 30 μg/mL pepstatin A. However, the viability of the cells in the presence of pepstatin was still below that of the controls, an indication that protection by inhibiting cathepsin D was not complete. These data suggest that GAELs may induce a cell death pathway involving acid proteases that contribute to the overall loss of cell viability in some primary cell samples, but this is not a universal response of EOC cells to MO-101 and is likely not the only means by which MO-101induces cell death.

**Fig. 6:**
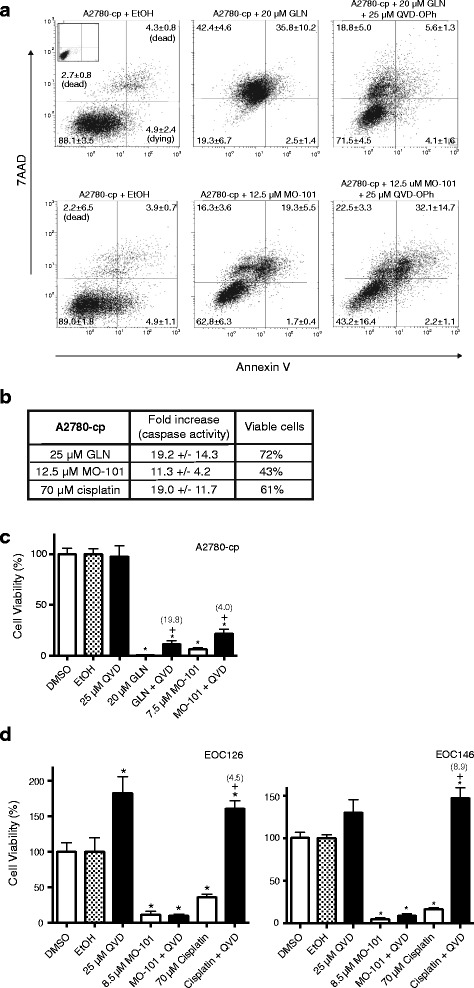
Examining the role of caspases for GAEL-induced cell death. **a** Viability of A2780-cp cells treated with GAELs alone or pretreated with the pan-caspase inhibitor Q-VD-OPh (QVD) for 4 h, prior to co-treatment with GAELs and QVD for 48 h. Percentage of cells in each quadrant are indicated. **b** Activated caspases were measured in A2780-cp cells 24 h after initiation of treatment with GLN, MO-101, or cisplatin. Fold caspase activity in comparison to vehicle control treated cells is indicated. Percent viability at the time of cell harvest is indicated as measured by trypan *blue* exclusion. **c** Viability of A2780-cp cells treated with GAELs alone or pretreated with the pan-caspase inhibitor Q-VD-OPh (QVD) for 4 h, prior to co-treatment with GAELs and QVD for 48 h. The fold increase in cell viability is shown in co-treated cells, indicated in brackets above the bar. * significantly different from vehicle control; + significantly different from GAEL alone; *p* < 0.005. **d** Viability of primary EOC cell samples, EOC126 or EOC146, treated with GAELs alone or pretreated with the pan-caspase inhibitor QVD. No significant difference in cell viability was observed in the presence of QVD, with the exception of the cisplatin positive control. * significantly different from vehicle control; + significantly different from cisplatin alone; *p* < 0.005

Although previous studies reported that GAELs did not kill cells by inducing apoptosis, the inability of pepstatin to attenuate GAEL effects in A2780-cp cells, led us to investigate whether activation of apoptosis could play a role in GAEL-induced cell death in these cells. A2780-cp cells were incubated with GLN or MO-101 and cell death was quantified with annexin V plus 7AAD staining. The results displayed in Fig. 6a show a dose-dependent increase in Annexin V positive dying and dead cells in response to GLN and MO-101 treatment for 48 h. The highest doses of GLN (25 μM) and MO-101 (12.5 μM) resulted in up to 78% (upper quadrants, 42.4% + 35.8%) or ~36% (16.3% + 19.3%) dead cells, respectively. The presence of dying/dead cells that stained for Annexin V suggested that GAELs may induce apoptosis in A2780-cp cells. To further investigate this, caspase activation in cells treated with GAELs was assessed. A2780-cp cells were treated for 48 h with 25 μM GLN, 12.5 μM MO-101, or 70 μM cisplatin (as a positive control to induce caspase activity) and caspase activity was measured using the Caspglow assay. The results showed that there was an increase in caspase activity in response to each treatment (Fig. 6b). The percentage of viable cells at the conclusion of each experiment as determined by trypan blue dye exclusion assay revealed the GAEL-induced increase in caspase activity corresponded with a reduction in viable cells.

In order to test whether caspase activity was linked with the reduction in cell viability, A2780-cp cells were treated with GAELs in the presences of the pan-caspase inhibitor Q-VD-OPh (QVD). A2780-cp cells were pre-treated with or without 25 μM QVD for 4 h prior to the addition of the GAEL. In the GLN + QVD treated cells there was a 3.2-fold decrease in the number of dead cells compared to GLN-alone (Fig. 6a). In similar experiments conducted with non-adherent aggregate cultures, the percentage of viable cells as assessed by the CellTiter assay rose from 1% to about 20% in A2780-cp cells treated with GLN + QVD compared to GLN alone (Fig. 6c). Conflicting data was observed for MO-101 treated cells. In the presence of MO-101 + QVD, there was a 1.5 fold further increase in cell death compared to MO-101 alone (Fig. 6a). However, QVD reduced the amount of cell death induced by MO-101 using the CellTiter assay (Fig. 6c). These results support the notion that caspase activation occurs in A2780-cp cells in response to GAEL treatment, but pathways activated by the caspases are not solely responsible for cell death.

We next investigated whether inhibition of caspase activity in primary EOC cells would rescue the cells from GAEL-induced cell death using the approaches described above. Studies with EOC126 or EOC146 cells showed that pre-incubation with QVD prior to the addition of MO-101 did not result in an increase in viability relative to cells incubated with MO-101 alone (Fig. 6d; GLN was not tested on the primary EOC cell samples for this assay). Similar results were obtained for EOC183A (Additional file 8: Figure S7). It was notable that QVD treatment alone was capable of increasing the number of viable cells in EOC126 and EOC146, suggesting that there is a basal level of activated caspases in the EOC cells. As a positive control for these experiments, the effect of QVD on cisplatin-induced cell death, which is known to occur via caspase activation, was investigated. Cisplatin-induced caspase activity was efficiently blocked by pre-treatment with QVD (Fig. 6d and Additional file 8: Figure S7). Thus, in primary EOC cells GAEL-induced cell death appeared to be completely caspase-independent.

## Discussion and conclusions

There is an urgent need for novel drugs to effectively treat recurrent, drug-resistant EOC. Herein we show that GAELs effectively kill platinum-sensitive and platinum-resistant EOC cell lines and primary EOC cell samples in adherent and non-adherent culture systems. Cells grown as non-adherent cultures typically represent a more drug-resistant profile compared to monolayer cultures [9, 14–16]. The non-adherent culture conditions approximate cells growing as ascites in vivo. It was therefore important to test the cell killing effect of the anticancer drugs in vitro using the non-adherent culture conditions to obtain a better idea of possible responses in vivo. The data reported herein suggest that GAELs may constitute a novel drug class with potential for effectively treating different EOC histotypes, and more importantly platinum-resistant cells commonly found with recurrent EOC.

Our work confirms earlier reports showing enhanced cisplatin resistance of EOC cells when grown as non-adherent cultures relative to cell monolayers [9, 14, 15, 34], with some non-adherent cultures showing resistance to cisplatin up to the maximum dose tested (90 μM). By contrast, we showed that GAELs are effective at killing EOC cells grown as adherent monolayers, non-adherent cellular aggregates, or as spheroids at much lower concentrations. Depending on the assay used, this required exposure to the drugs for different time courses (48 h to test cell viability; 72–96 h when examining spheroid dissolution). Notably, the doses of GAELs required to kill EOC cells were a fraction of the cisplatin doses required to kill the same cells. GAELs effectively killed EOC cells from several primary HGSOC samples, a HGSOC cell line (COV362), as well as a primary clear cell EOC sample (EOC126), and an endometrioid EOC cell line (A2780).

For primary cells grown as non-adherent cultures, the dose of cisplatin needed to kill these cells was not attained within the dose range used, but were in excess of those required to kill the A2780-cp cisplatin resistant cell line. Relative resistance to MO-101 was observed in EOC126 and resistance to GLN in EOC140, EOC146, and EOC183 primary cell cultures. However, it is worth pointing out that the highest concentration of GAEL tested was 10 μM, and therefore it remains possible that complete cell death may have been achieved at higher concentrations. The reason why the different cells are susceptible to one GAEL and not the other is unclear but may reflect an inherent property of the EOC histotype (i.e. EOC126 are clear cell; the other primary samples are high grade serous), or genetic changes within that patient sample. GLN and MO-101 are structurally similar but are differentially charged as a consequence of the additional amino group on MO-101 relative to GLN. Noteworthy is the fact that all the EOC cells were susceptible to one GAEL or the other.

Experiments were conducted to investigate potential mechanism(s) of action for GAEL-induced cell death. Previous reports showed that GAELs induce a caspase-independent form of cell death, part of which was due to the release of acid proteases such as cathepsin D from the lysosomes into the cytosol [23, 28]. While pepstatin A, an inhibitor of cathepsin D, did not affect GAEL-induced death in A2780-cp, EOC140, or EOC183A cells, a protective effect was observed in EOC146 cells. This suggests that the involvement of cathepsin D to cell death may occur in some EOC patient samples, but is not be a universal event in all EOC cells.

In A2780-cp cells, GAELs induced the activation of caspases, and the increased caspase activity that correlated with reduced cell viability was partially blocked by the pan-caspase inhibitor (QVD). This was consistent for GLN, but MO-101 produced contrasting results. The presence of QVD had no effect on MO-101-induced cell death, and in fact enhanced the amount of cell death, when tested using flow cytometry. However, there was a reduction in cell death when tested using the CellTiter assay. These different results may be a reflection of the sensitivity of the different assays to the level of cell death, or it may reflect the different culture condition used for each assay. CellTiter experiments were conducted using a 96-well format, whereas the flow cytometry experiments required generation of cell aggregates and drug treatment in several wells of a 12-well plate. The flow cytometry experiments required a greater number of cells, and thus several wells were pooled for a single condition.

GLN and MO-101-induced cell death appears to be partially dependent on caspase activity in A2780-cp cells. By contrast, the presence of QVD had no effect on MO-101-induced death in primary EOC cells. These results are similar to our previous results using different cancer cell types and other GAELs [28]. Thus, in EOC cells, GAEL-induced cell death is primarily via a caspase-independent mechanism that may be augmented by caspase activation in A2780-cp cells. One beneficial effect of this is that if the apoptosis pathway is inhibited in the cancer cell, cell death may still occur via the caspase-independent mechanisms. Additional research is required to illuminate the mechanisms of cell death induced by GAEL compounds. The overall conclusion from our studies is that GAELs in general, and MO-101 in particular, will be an excellent lead agent for in vivo assessment of its efficacy against platinum-resistant ovarian cancer. While in vivo studies have not been conducted with these compounds, future studies are planned to investigate the tolerability and efficacy of GAELs, and MO-101 in particular, for treating EOC in vivo.

## Additional files

Additional file 1: Table S1. Primary EOC patient sample histological diagnosis, surgical staging and/or chemotherapy resistance status, and CA-125 levels at time of cell sampling.

Additional file 2: Figure S1. Chemical structure of GLN and MO-101. Note the amino substitution at the C_6_-position of glucose in MO-101.

Additional file 3: Figure S2 a. Images of ovarian cancer cell clusters present in ascites fluid from patients EOC174 and EOC189. EOC174 had a larger number of cell clusters and larger clusters resembling spheroids. **b**. Representative images of EOC cells (A2780-cp cell line or primary cell samples EOC140 and EOC146) grown as adherent monolayers, non-adherent aggregates or spheroids at 2 and 7 days after seeding ultralow attachment plates. EOC140 are capable of forming spheroids by 7 days after seeding, whereas EOC146 formed numerous spheroid structures within 2 days.

Additional file 4: Figure S3. Effect of MO-101 on spheroid integrity. Spheroids from EOC013F and EOC061 were exposed to increasing doses of MO-101. Spheroid integrity was observed after 72 h of drug exposure. Spheroid disintegration is observed with increasing doses. The grainy material in some panels is cellular debris.

Additional file 5. Figure S4. Drug sensitivity of COV362 HGSOC cell line, and three primary HGSOC patient samples. Cisplatin dose–response curves for COV362, EOC146, EOC183A, EOC183I cells grown as adherent (closed circle) or non-adherent (open square) cultures. Intersections with the dashed line approximates the CC_50_ value. Drug doses where there is a significant difference in cell viability between culture conditions or treatments are indicated by an asterisk (*), *p* < 0.05.

Additional file 6: Figure S5. Dose–response curves for COV362 cells and three primary HGSOC patient samples (EOC146, EOC183A, EOC183I) to GLN (open circle) or MO-101 (closed diamond). Intersections with the dashed line approximates the CC_50_ value. Drug doses where there is a significant difference in cell viability between culture conditions or treatments are indicated by an asterisk (*), *p* < 0.05.

Additional file 7: Figure S6. Evaluating the ability of pepstatin A (PepA) to block GAEL-induced cell death in EOC140 cells. **a**. EOC183A cells were treated with increasing doses of MO-101 alone or co-treated with 25 μg/mL PepA for 48 h. While MO-101 or MO-101 + PepA treatment induced a significant level of cell death, there was no statistically significant rescue from cell death in the presence of PepA. **b**. EOC146 cells were treated with 5 μM MO-101 alone or co-treated with 20 or 30 μg/mL PepA for 48 h. There was a statistically significant increase in cell viability for cells treated with MO-101 + 30 μg/mL PepA compared to MO-101 alone (+). For both A and B doses where there is a significant difference in cell viability between cells grown in the presence of drugs compared to vehicle control or PepA alone are indicated by asterisks (***), *p* < 0.001.

Additional file 8: Figure S7. Viability of primary EOC183A cells treated with GAELs alone or pretreated with the pan-caspase inhibitor QVD. No significant difference in cell viability was observed in the presence of QVD, with the exception of the cisplatin positive control. * significantly different from vehicle control; + significantly different from cisplatin alone; *p* < 0.005.
