# Supplementary material for: Novel glycolipid agents for killing cisplatin-resistant human epithelial ovarian cancer cells
Source: J Exp Clin Cancer Res. 2017 May 12;36:67. doi: 10.1186/s13046-017-0538-9 (PMC5429581; doi:10.1186/s13046-017-0538-9)
Supplement: Supplementary file 1 — Primary EOC patient sample histological diagnosis, surgical staging and/or chemotherapy resistance status, and CA-125 levels at time of cell sampling. [file 13046_2017_538_MOESM1_ESM.pdf]

Supplementary Material

Supplemental Table 1. Primary EOC patient sample histological diagnosis, surgical staging and/or chemotherapy resistance status, and CA-125 levels at time of cell sampling.

Sample	Histotype	Staging/Chemo status	CA-125 at time of sampling
**EOC013F**	Ovarian adenocarcinoma	Stage IIIC. High grade.	Not available
**EOC016B**	Ovarian adenocarcinoma	Chemonaïve	755
**EOC016H**	Ovarian adenocarcinoma	Recurrent, platinum resistant; sample obtained 10 weeks after EOC16B	4560
**EOC058**	Ovarian high grade serous adenocarcinoma	Stage IIIC; Chemonaïve	284
**EOC061**	Ovarian high grade serous adenocarcinoma	Not available	Not available
**EOC126**	Ovarian clear cell adenocarcinoma	Stage II; Chemonaïve	977
**EOC140**	Ovarian high grade serous cystadenocarcinoma	Stage IIIC; recurrent, platinum resistant	50
**EOC146**	Ovarian high grade serous cystadenocarcinoma	Stage IIIC; recurrent, platinum resistant	787
**EOC183A**	Ovarian high grade serous adenocarcinoma	Stage IIIC; recurrent, platinum resistant	469
**EOC183I**	Ovarian high grade serous adenocarcinoma	Stage IIIC; recurrent, platinum resistant	376
