# Supplementary figures and images for: Novel glycolipid agents for killing cisplatin-resistant human epithelial ovarian cancer cells
Source: J Exp Clin Cancer Res. 2017 May 12;36:67. doi: 10.1186/s13046-017-0538-9 (PMC5429581; doi:10.1186/s13046-017-0538-9)

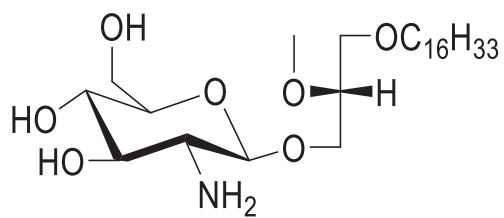

$\beta$ -GLN

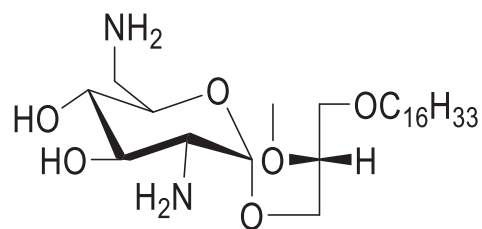

MO-101

Supplement: Supplementary file 2 — Chemical structure of GLN and MO-101. Note the amino substitution at the C6-position of glucose in MO-101. [file 13046_2017_538_MOESM2_ESM.pdf]

EOC cells direct from ascites  
(arrows indicate cell aggregates)

Supplemental Figure 2

**A.**

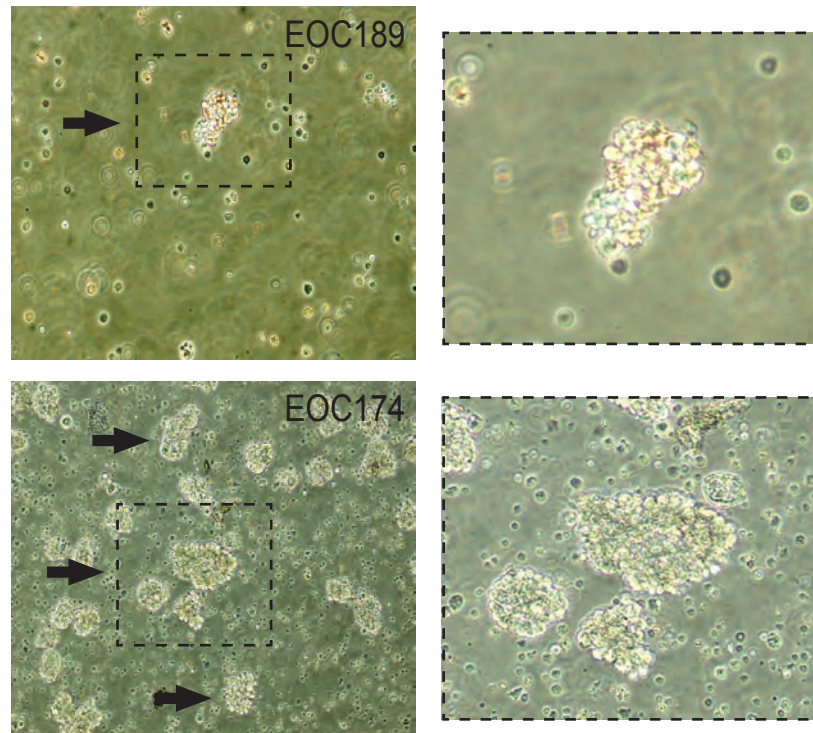

**B.**

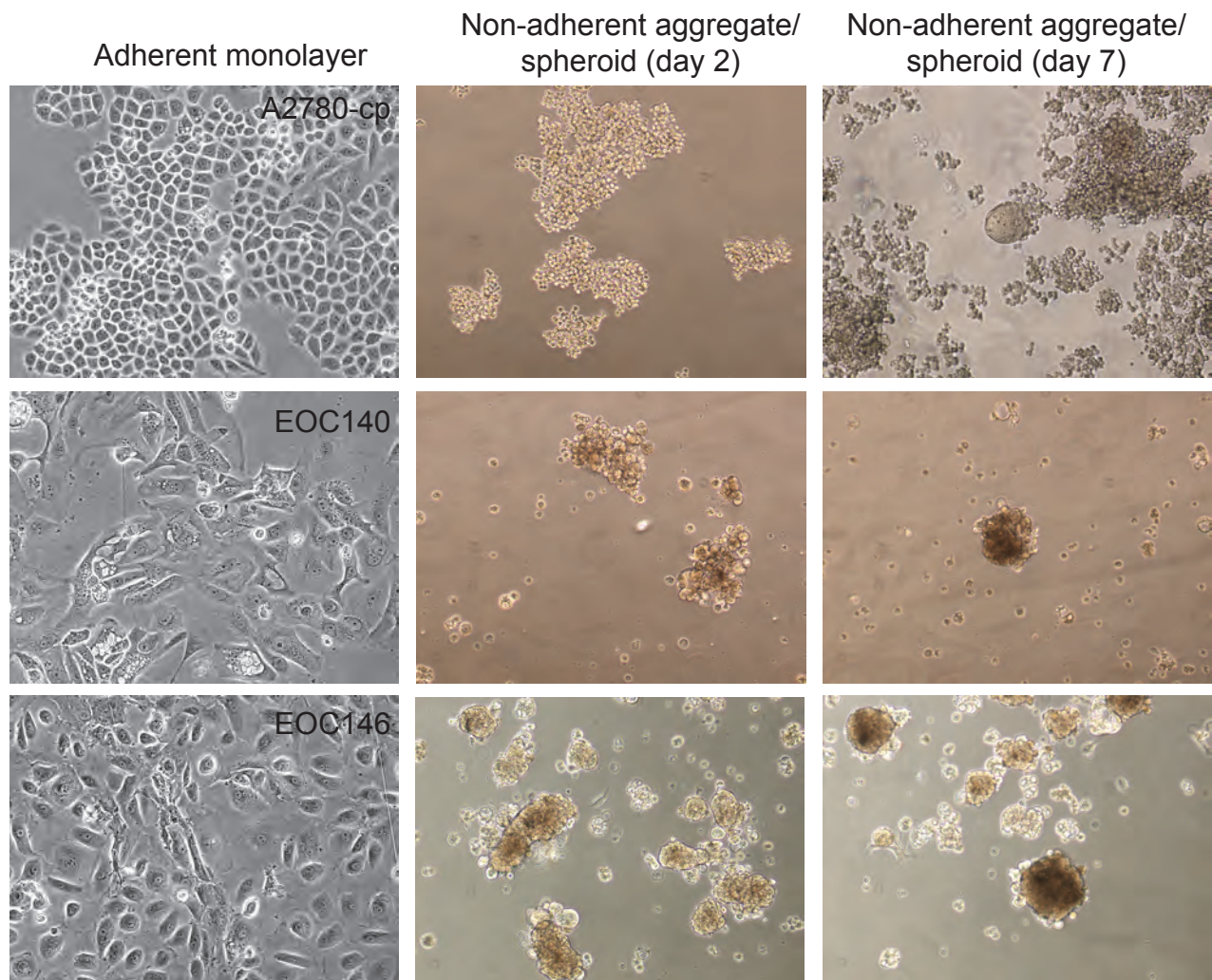

Supplement: Supplementary file 3 — Images of ovarian cancer cell clusters present in ascites fluid from patients EOC174 and EOC189. EOC174 had a larger number of cell clusters and larger clusters resembling spheroids. b. Representative images of EOC cells (A2780-cp cell line or primary cell samples EOC140 and EOC146) grown as adherent monolayers, non-adherent aggregates or spheroids at 2 and 7 days after seeding ultralow attachment plates. EOC140 are capable of forming spheroids by 7 days after seeding, whereas EOC146 formed numerous spheroid structures within 2 days. [file 13046_2017_538_MOESM3_ESM.pdf]

EOC013F - 72 hours

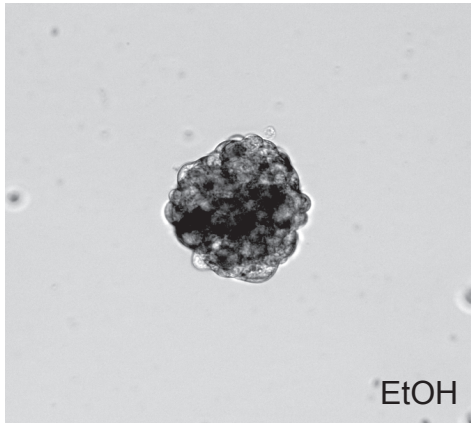

EOC061 - 72 hours

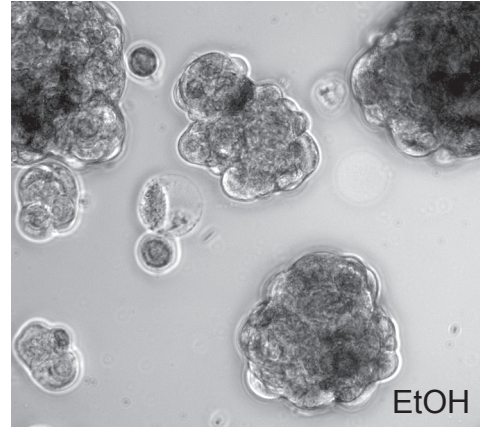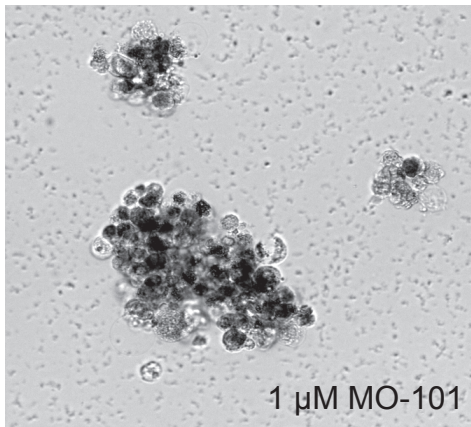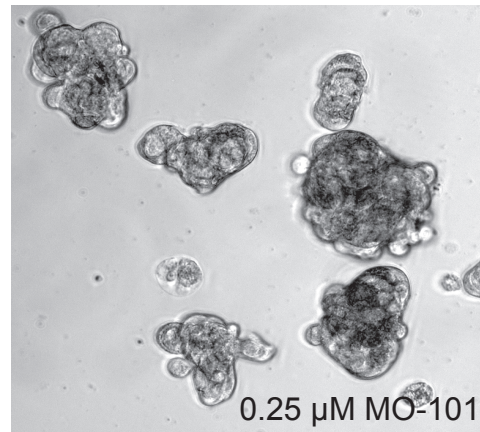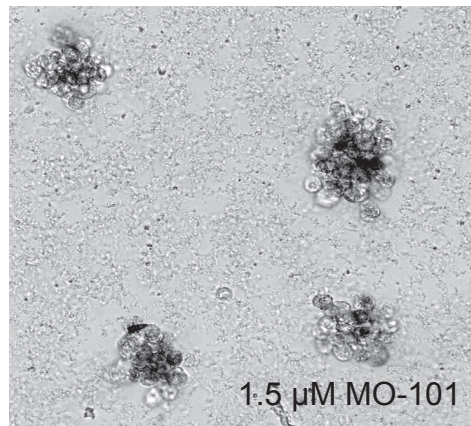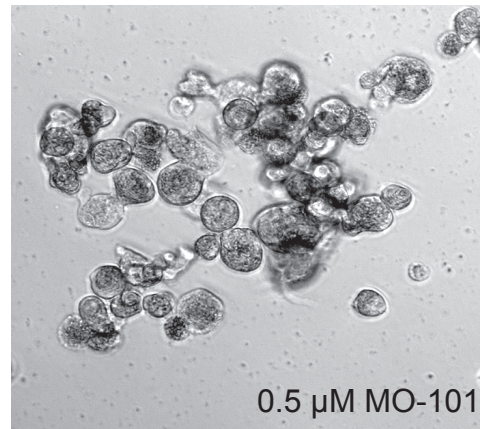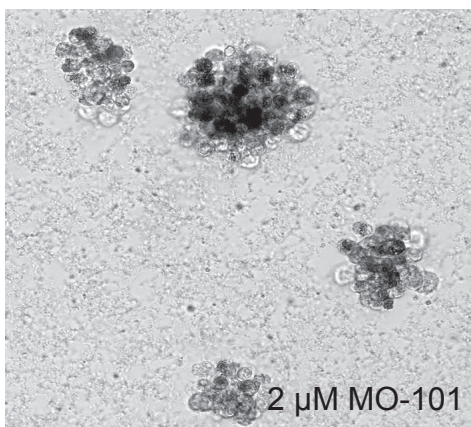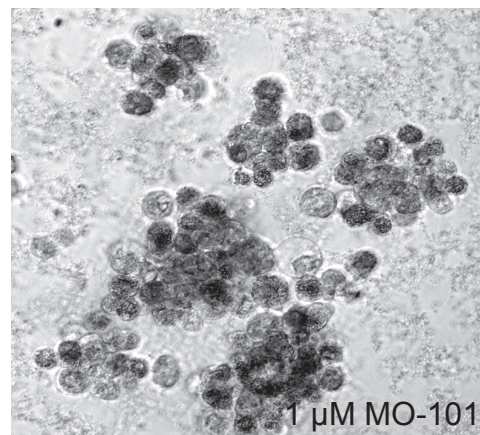

Supplement: Supplementary file 4 — Effect of MO-101 on spheroid integrity. Spheroids from EOC013F and EOC061 were exposed to increasing doses of MO-101. Spheroid integrity was observed after 72 h of drug exposure. Spheroid disintegration is observed with increasing doses. The grainy material in some panels is cellular debris. [file 13046_2017_538_MOESM4_ESM.pdf]

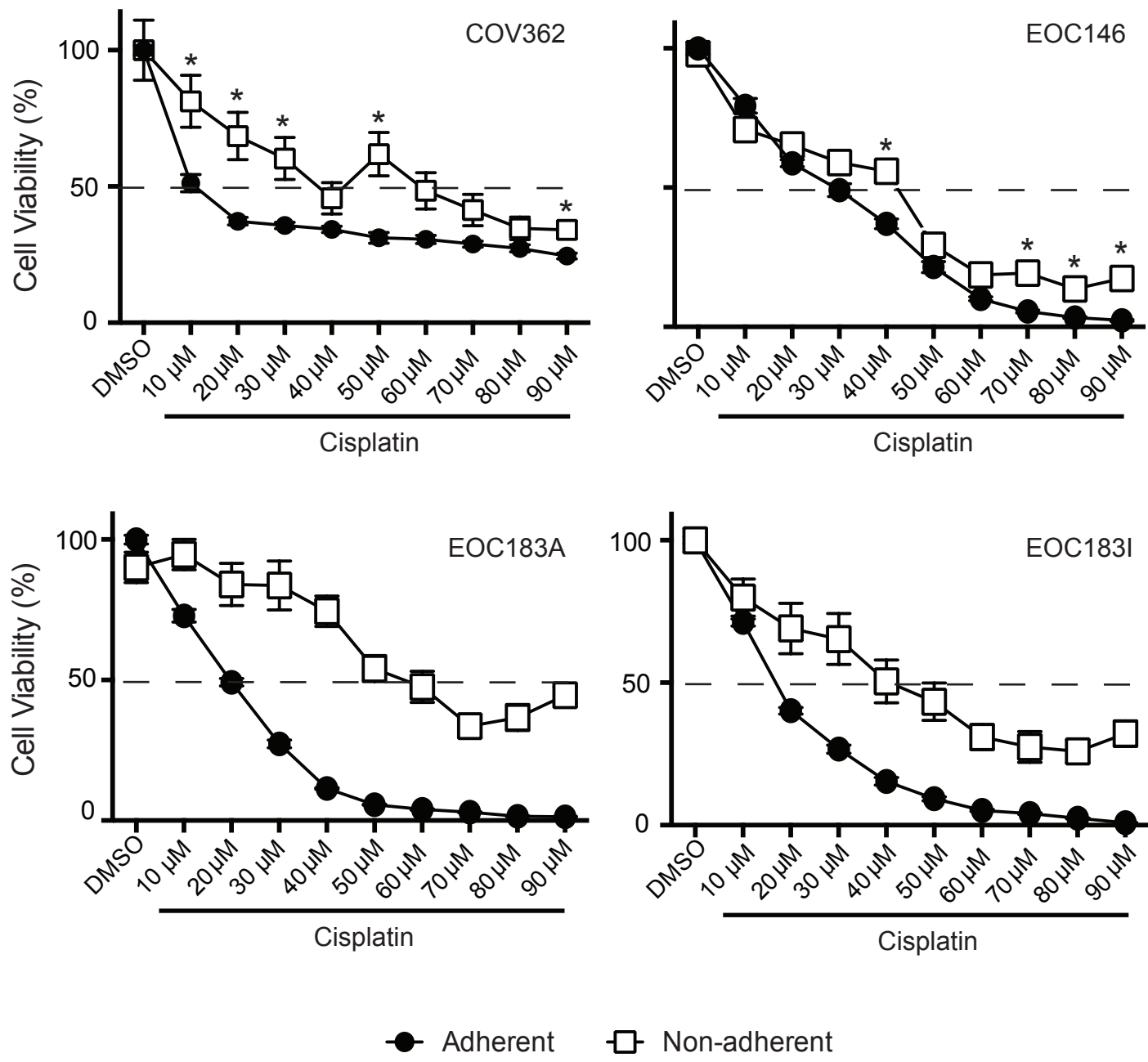

Supplement: Supplementary file 5 — Drug sensitivity of COV362 HGSOC cell line, and three primary HGSOC patient samples. Cisplatin dose–response curves for COV362, EOC146, EOC183A, EOC183I cells grown as adherent (closed circle) or non-adherent (open square) cultures. Intersections with the dashed line approximates the CC50 value. Drug doses where there is a significant difference in cell viability between culture conditions or treatments are indicated by an asterisk (*), p < 0.05. [file 13046_2017_538_MOESM5_ESM.pdf]

Supplemental Figure 5

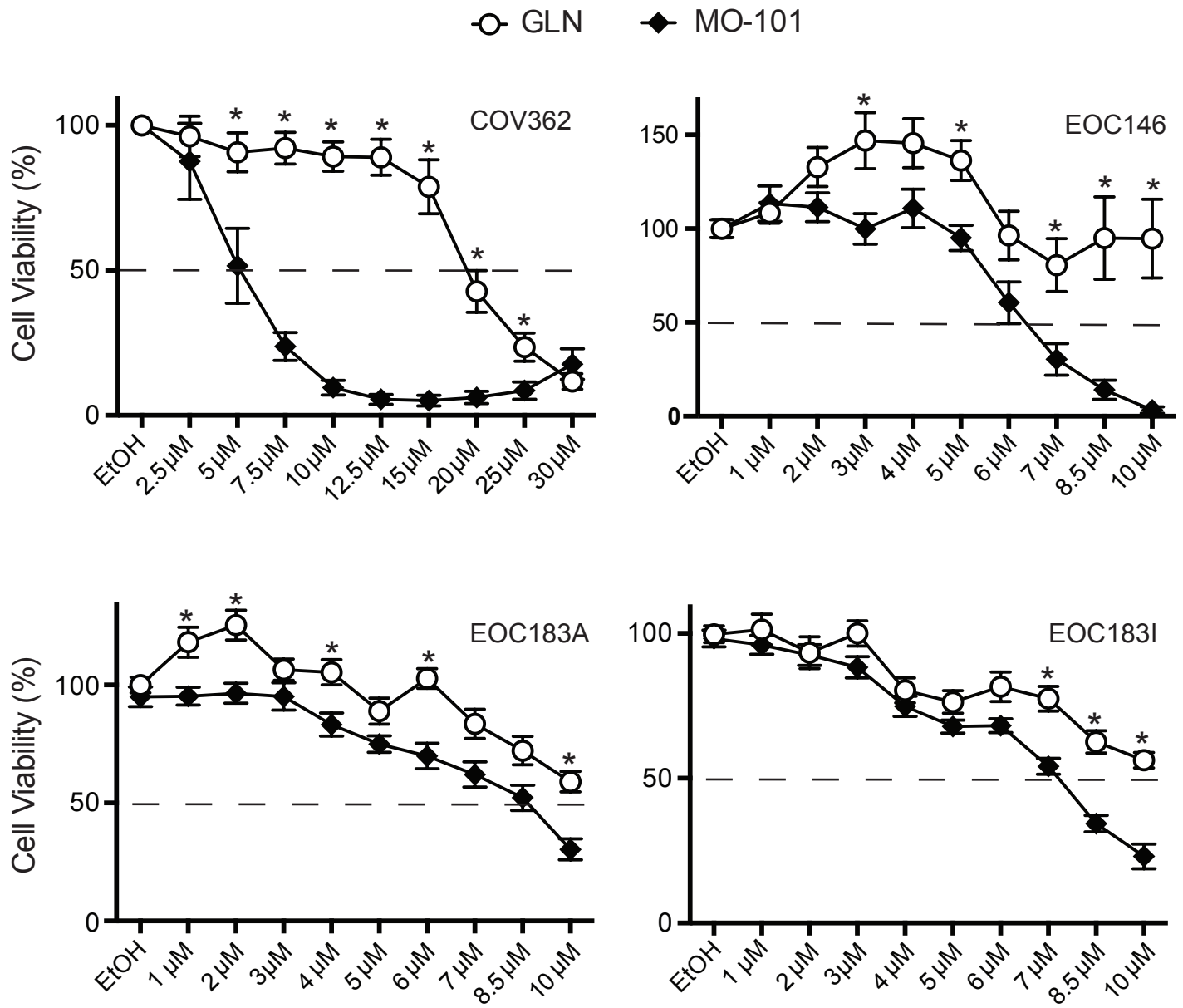

Supplement: Supplementary file 6 — Dose–response curves for COV362 cells and three primary HGSOC patient samples (EOC146, EOC183A, EOC183I) to GLN (open circle) or MO-101 (closed diamond). Intersections with the dashed line approximates the CC50 value. Drug doses where there is a significant difference in cell viability between culture conditions or treatments are indicated by an asterisk (*), p < 0.05. [file 13046_2017_538_MOESM6_ESM.pdf]

**A.**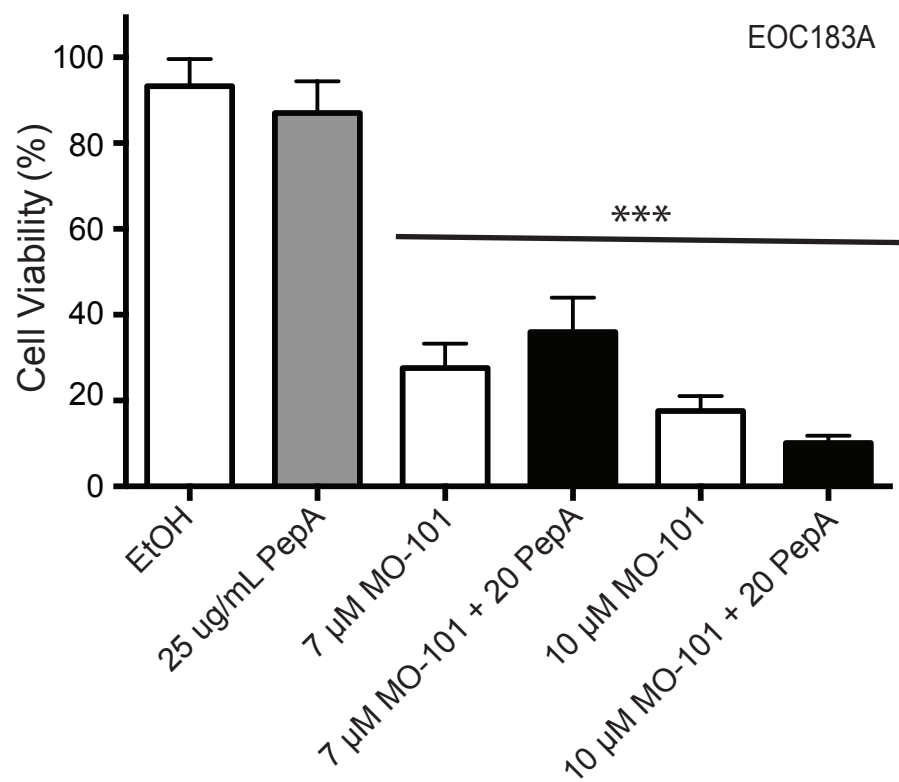**B.**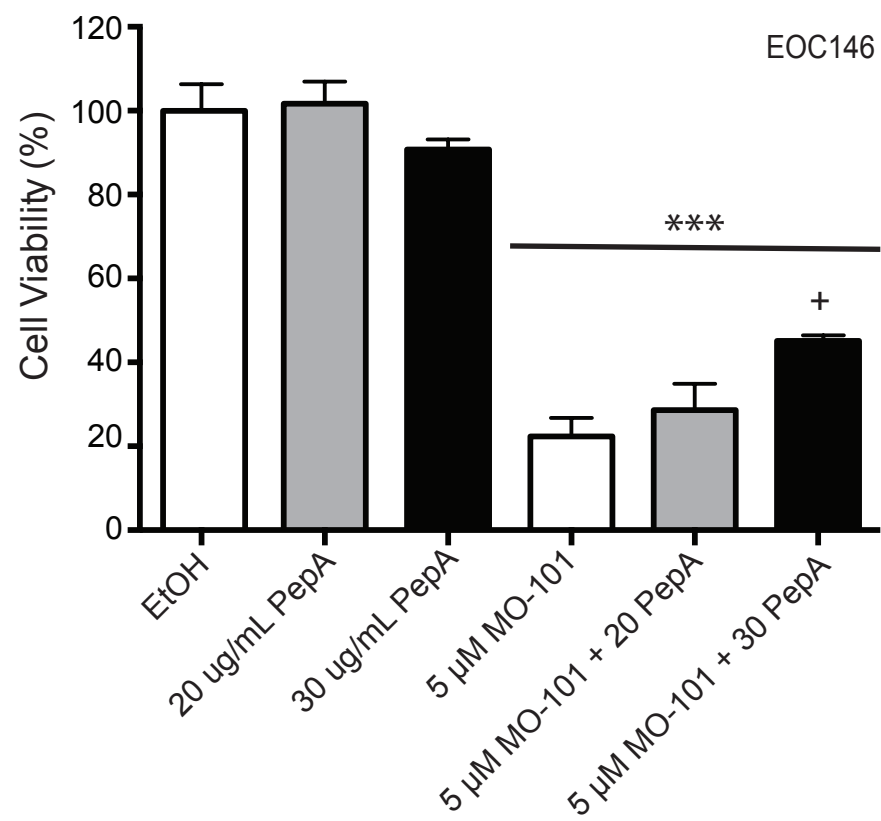

Supplement: Supplementary file 7 — Evaluating the ability of pepstatin A (PepA) to block GAEL-induced cell death in EOC140 cells. a. EOC183A cells were treated with increasing doses of MO-101 alone or co-treated with 25 μg/mL PepA for 48 h. While MO-101 or MO-101 + PepA treatment induced a significant level of cell death, there was no statistically significant rescue from cell death in the presence of PepA. b. EOC146 cells were treated with 5 μM MO-101 alone or co-treated with 20 or 30 μg/mL PepA for 48 h. There was a statistically significant increase in cell viability for cells treated with MO-101 + 30 μg/mL PepA compared to MO-101 alone (+). For both A and B doses where there is a significant difference in cell viability between cells grown in the presence of drugs compared to vehicle control or PepA alone are indicated by asterisks (***), p < 0.001. [file 13046_2017_538_MOESM7_ESM.pdf]

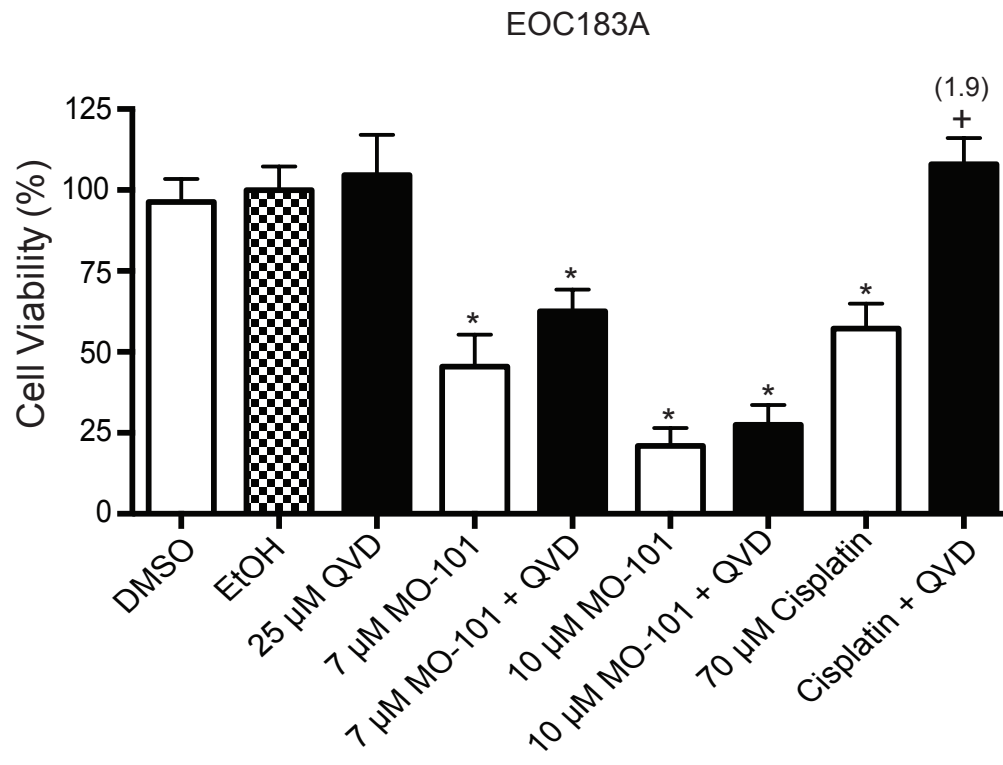

Supplement: Supplementary file 8 — Viability of primary EOC183A cells treated with GAELs alone or pretreated with the pan-caspase inhibitor QVD. No significant difference in cell viability was observed in the presence of QVD, with the exception of the cisplatin positive control. * significantly different from vehicle control; + significantly different from cisplatin alone; p < 0.005. [file 13046_2017_538_MOESM8_ESM.pdf]
